# Dried Biomass of *Arthrospira platensis* Inhibits Growth of *Aureobasidium pullulans* LW14 and Some Bacteria When Added to Unpasteurised Apple Juice

**DOI:** 10.1007/s12088-020-00871-w

**Published:** 2020-04-18

**Authors:** Łukasz Wajda, Zuzanna Rękas, Tomasz Tarko, Aleksandra Duda-Chodak, Aleksandra Liebersbach, Małgorzata Makarewicz

**Affiliations:** 1 Malopolska Centre of Biotechnology, Jagiellonian University, Gronostajowa 7A str, 30-387 Kraków, Poland; 2 Department of Fermentation Technology and Technical Microbiology, Faculty of Food Technology, University of Agriculture in Krakow, ul. Balicka 122, 30-149 Kraków, Poland

**Keywords:** Food preservation, Fungi, Lactic acid bacteria, Cyanobacteria, Antimicrobials

## Abstract

The main goal was to evaluate if dried cyanobacterium (*Arthrospira platensis*) biomass added to unpasteurised apple juice could inhibit growth of fungi dominant in apples and which fraction of cyanobacterium biomass contributed to this phenomenon. *A. platensis* biomass, its protein and/or polysaccharide fraction were added to the sterilised apple juice inoculated with *Aureobasidium pullulans* LW14 and stored at 4 °C for 96 h. Samples were tested against number of fungi and selected physical–chemical parameters. In the last research stage, protein fraction was added to the unpasteurised apple juice inoculated with *A. pullulans* LW14 to test its potency against fungi, lactic acid bacteria and acetic acid bacteria. When *A. platensis* biomass was added to the sterilised apple juice (1.15% w/v), it effectively inhibited growth of *A. pullulans* LW14 and the metabolism of all analysed sugars (glucose, sucrose and fructose). Protein fraction added to the sterilised apple juice contributed significantly to mycostatic properties of *A. platensis*. When protein fraction was added to unpasteurised apple juice, the same conclusions were obtained, however, except for inhibiting growth of *A. platensis* LW14, it inhibited growth of some bacteria.

## Introduction

The biomass of *A. platensis* holds antimicrobial properties. Pharmaceutically active extract made of the extrudate of the *A. platensis* inhibits growth of common human pathogens, i.e. *Propionibacterium acnes*, *Staphylococcus epidermis*, *S. aureus* etc. [1]. Yet, the data focusing on the influence of *A. platensis* biomass on microorganisms causing food spoilage is very scarce. According to our knowledge only one study [2] addressed that issue. Based on her findings, water extracts prepared from tablets containing *A. platensis* biomass inhibited growth of *Bacillus subtilis*, *Micrococcus luteus*, *Rhodotorula* sp. or *Penicillium* sp. and stimulated growth of *Alicyclobacillus acidoterrestris, Geotrichum* sp. or *Cladosporium* sp.

Inhibitory activity of Spirulina could be related to the presence of carotenoids coupled with protein [3] since carotenoids demonstrate antibacterial and antifungal properties [4] and their content in *A. platensis* biomass reaches at least 4 mg g day/m [5]. Antimicrobial properties were also demonstrated for phycobiliprotein which content in *A. platensis* biomass is up to 14% [6]. Both these groups of substances are water soluble which means that they dissolve in an apple juice. Another group of water-soluble substances in cyanobacterium biomass are polysaccharides. It has been already demonstrated that some of them hold antiviral properties [7]. There are also other bioactive compounds of *A. platensis* biomass that participate in antimicrobial effect but they were not considered in the current study since they dissolve in organic solvents, i.e. chitinase, chlorophylls or fatty acids [8].

Preliminary experiments demonstrated that the addition of *A. platensis* biomass to the unpasteurised apple juice sustained total viable microbial count for the whole storage period [9]. In the current study we narrowed down the dose of *A. platensis* biomass in an apple juice which could inhibit fungal growth and determined which fraction of cyanobacterium biomass participated in this phenomenon. For that purpose we carried out experiments in sterilised apple juice inoculated with *Aureobasidium pullulans* LW14 that was the representative of the species dominating fungal microflora and which demonstrated the highest synthesis of exopolysaccharides [10]. In the last research stage we verified obtained results by adding selected cyanobacterium fraction to the unpasteurised apple juice.

## Materials and Methods

Microbiological media were purchased from BIOCORP (Gliwice, Poland) and spray-dried biomass of *Arthrospira platensis* (Ivarsson’s Hawaian Spirulina) was manufactured by Ivarsson’s Produkte für’s Leben (Schriesheim, Germany). Apples (Koksa Górska cultivar) were obtained from the private orchard in Łososina (Poland). All other chemicals were purchased from Dor-Chem (Kraków, Poland), unless otherwise stated.

### Pre-treatment of *Arthrospira platensis* Biomass

The biomass of *Arthrospira platensis* was combined with ethanol (1:1 ratio) and homogenised (2 min 19,000/rpm) with Ultra Turrax T-25-basic homogeniser (IKA-Werke GmbH & Co. KG, Staufen, Germany). Ethanol was evaporated with carbon dioxide to dryness under aseptic conditions and obtained product will be further referred to as ‘Spirulina’. Spirulina was kept in dark at 4 °C till further experiments up to 4 h.

Phycocyanin was isolated according to the previously described procedure [11]. Exopolysaccharides were extracted as per Trabelsi et al. [12]. Protein and exopolysaccharides were lyophilised (Alpha 1–2 LD plus lyophiliser, Martin Christ Gefriertrocknungsanlagen GmbH, Osterode am Harz, Germany) overnight and kept at 4 °C for further experiments.

### Selection of Spirulina concentration

Fruits of Koksa Górska were harvested in 2017 in the middle of October, washed and cut into smaller pieces. Apple juice was obtained with the home juicer MPM J-45 (MPM AGD S.A., Milanówek, Poland), filtered through the cheese cloth and the half of its volume was frozen. The rest of fresh juice was used for the preparation of four experimental variants: control sample and three suspensions of Spirulina (1.15, 1.30 and 1.45% w/v) in 100 ml in capped bottles. They were stored for 96 h at 4 °C and the number of fungi was assessed each 24 h using WL nutrient agar with 100 mg of chloramphenicol/l. In our previous research [9] we demonstrated that Spirulina concentration exceeding 1% could effectively inhibit microbial growth, therefore, we decided to estimate specific concentration to achieve that effect against fungi.

### Antifungal Activity of Spirulina Fractions Against *A. pullulans* LW14 in Model Solution (Sterilised Apple Juice)

Frozen apple juice was thawed and sterilised after 14 days of storage. We used sterilised juice to exclude the impact of the native microflora. *Aureobasidium pullulans* LW14 (GenBank accession no. MG669476) was inoculated in sterilised juice and incubated on the rotary shaker (Orbit 1000, Labnet International Inc., Edison, NJ, USA) at 120 rpm for 24 h before the experiment. Cell number of LW14 strain was adjusted to approximately 10^4^ cells/ml with Thoma cell counting chamber. We prepared five experimental variants: sterilised apple juice inoculated with 10% (v/v) of LW14 suspension (FJA); sterilised apple juice inoculated with 10% (v/v) of LW14 suspension with 1.15% of Spirulina w/v (FJAS); sterilised apple juice inoculated with 10% (v/v) of LW14 and protein fraction (FJAPt); sterilised apple juice inoculated with 10% of *A. pullulans* LW14 suspension and polysaccharide fraction F(JAPl); sterilised apple juice inoculated with 10% of *A. pullulans* LW14 and both protein and polysaccharide fractions (FJAPP). The concentration of protein fraction was 0.72% (w/v) while the concentration of polysaccharide was 0.07% (w/v). In previous experiment it was demonstrated that 1.15 g of Spirulina in 100 ml of apple soft drink effectively inhibited growth of *A. pullulans* LW14. It was determined that 1.15 g of Spirulina contained 0.72 g of protein measured with Lowry method [13] and 0.07 g of polysaccharide measured with the method of Trabelsi et al. [12].

### Antifungal activity of protein from Spirulina in real solution (unpasteurised apple juice)

In the last research stage, there were four experimental variants: unpasteurised apple juice (UJ); unpasteurised apple juice with 10% (v/v) of LW14 suspension (UJA); unpasteurised apple juice with 10% (v/v) of LW14 suspension with 1.15% Spirulina w/v (UJAS); unpasteurised apple juice with 10% (v/v) of LW14 suspension with 0.72% protein fraction w/v (UJAP). Initial cell number of LW14 strain was 10^3^ cells/ml. All variants were stored at 4 °C for 96 h. We determined total viable count [14], the number of acetic acid bacteria (AAB) using calcium carbonate agar [15], the total number of fungi using WL nutrient agar with chloramphenicol. Enumeration of lactic acid bacteria was carried out with modified ISO method [16]—MRS agar was supplemented with 20% (v/v) of sterile apple juice and 10 ml of sorbic acid solution (1.4 g/10 ml of 1 M NaOH) per litre.

### Physical–Chemical Analyses

Samples were centrifuged at 14,000×*g*/5 min. Then 0.2 ml of clear supernatant was diluted with distilled water to 2 ml and filtered through syringe filters. The analysis was carried out with NEXERA XR chromatograph equipped with the refractometric detector RF-20A (Shimadzu, Sapporo, Japan). The separation of tested compounds was carried out on the Asahipak NH2P-50 250 × 4.6 mm Shodex column (Showa Denko Europe, Germany) which was thermostated at 30 °C. A 70% aqueous solution of acetonitrile was used as a mobile phase and elution was carried out isocratically for 20 min at 0.8 ml/min flow rate. Fructose, glucose, sucrose, sorbitol and glycerol were used for the preparation of calibration curves. Total acidity was measured as described in OIV methods [15] and expressed as g of malic acid/l. We used pH-meter to determine pH. Samples in experiments involving Spirulina fractions were collected each 24 h.

### Statistical analysis

All experiments were carried out in five replicates. The results were shown as arithmetic mean ± standard deviation. The normality of data distribution was assessed with Shapiro–Wilk test and the significance of experimental parameters was assessed with two-way analysis of variance. The significance of differences between means was assessed with post hoc Tukey test. All statistical analyses were carried out in R: A language and environment for statistical computing, version 3.3.1 (Foundation for Statistical Computing, Vienna, Austria, 2016).

## Results and Discussion

### Antifungal Activity of Selected Fractions of Spirulina Against *A. pullulans* LW14 in Sterilised Apple Juice

The average shelf life of unpasteurised apple juice is up to 48 h (based on the analysis of products available in the market and conversations with professionals) so all experiments were carried out for 96 h to assess if the addition of Spirulina could enhance microbial stability of the obtained soft drink. According to the European law, after adding Spirulina to the fruit juice, resulting finished product must not be referred to as ‘juice’ [17], therefore, it will be called a ‘soft drink’ throughout the whole paper. Our previous studies [9] indicated that Spirulina concentration should be at least 1% (w/v) in apple soft drinks to inhibit microbial growth and food manufacturers recommend that it should not exceed 1.50% w/v [5]. Moreover, in our previous research [18] we demonstrated that Spirulina concentration in unpasteurised apple soft drinks should not exceed 1.5% due to negative changes of organoleptic properties. Therefore, in the current study we assessed minimum dose which prevents microbial growth within that range. All tested Spirulina concentrations (1.15, 1.30 and 1.45% w/v) effectively inhibited fungal growth in the unpasteurised apple soft drink (Table 1) for the whole storage time so further experiments were continued with 1.15% of Spirulina.

**Table 1:** Fungal growth in unpasteurised apple juice supplemented with Spirulina over the extended storage time

Day	Spirulina dose [%w/v]			
	0 [cfu/ml]	1.15 [cfu/ml]	1.30 [cfu/ml]	1.45 [cfu/ml]
0	2.18 ± 0.05 × 10^3a^	1.57 ± 0.18 × 10^3b^	1.75 ± 0.14 × 10^3b^	1.77 ± 0.08 × 10^3b^
1	> 3 × 10^3c^	1.45 ± 0.12 × 10^3b^	1.63 ± 0.17 × 10^3b^	1.77 ± 0.22 × 10^3b^
2	> 3 × 10^3c^	1.54 ± 0.20 × 10^3b^	1.34 ± 0.16 × 10^3b^	1.43 ± 0.11 × 10^3b^
3	> 3 × 10^4d^	1.30 ± 0.24 × 10^3b^	1.52 ± 0.41 × 10^3ab^	1.74 ± 0.52 × 10^3ab^
4	> 3 × 10^5e^	> 3 × 10^3c^	>3 × 10^3c^	2.30 ± 0.45 × 10^3b^

The same letters next to means indicate the lack of statistically significant differences at p < 0.05 in the whole table, n = 5

Next research steps aimed to explain the phenomenon of mycostatic properties of cyanobacterium biomass. To accomplish that goal, we used sterilised juice to exclude the impact of the native microflora. We adjusted the number of fungal cells to that which was found in freshly harvested apples. Previously we demonstrated that the most abundant species in apple of Koksa Górska variety was *Aureobasidium pullulans* so in the current study we chose *A. pullulans* LW14 strain that was proved to be potent exopolysaccharide producer [10] and could potentially spoil unpasteurised juice.

**Fig. 1:**
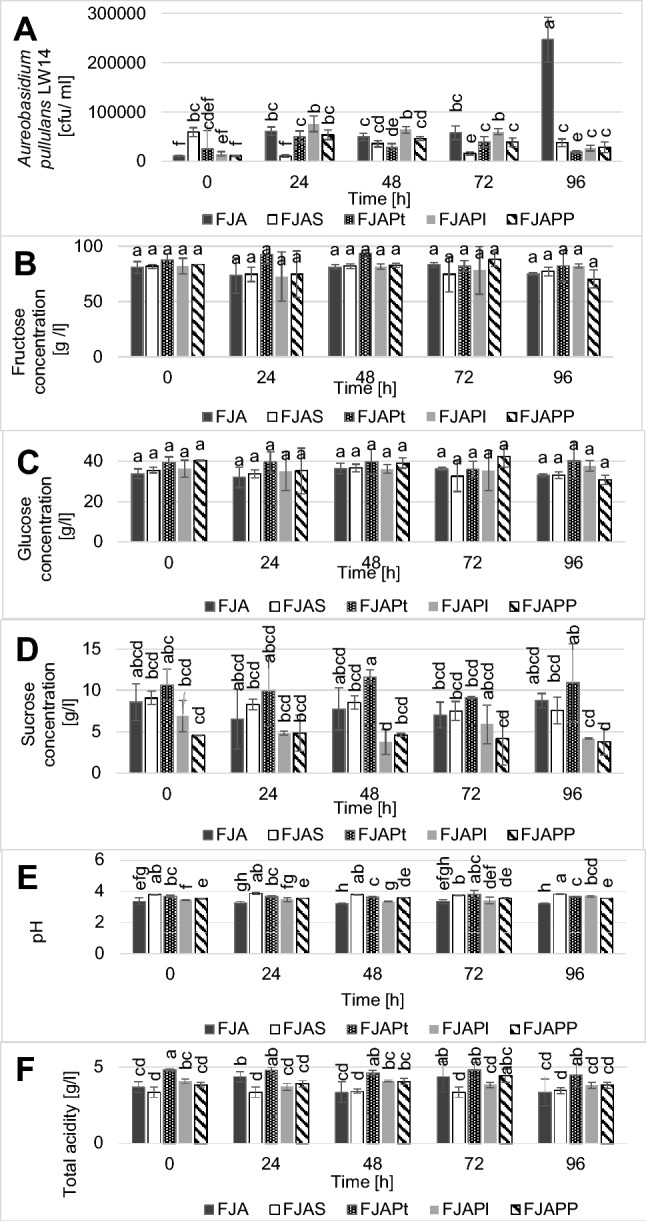
Microbiological, chemical and physical changes in sterilised apple juice fortified with selected Spirulina fractions: **a***Aureobasidium pullulans* LW14 growth; **b** fructose concentration; **c** glucose concentration; **d** sucrose concentration; **e** pH; **f** total acidity expressed as malic acid. The same letters above columns indicate the lack of statistically significant differences at: p < 0.05 (1f); p < 0.01 (1e); p < 0.001 (1a), p < 0.0001 (1d), n = 5. In case of 1b and 1c differences were not statistically significant. *FJA* sterilised apple juice + A. pullulans LW14, *FJAS* sterilised apple juice + *A. pullulans* LW14 + Spirulina, *FJAPt* sterilised apple juice + *A. pullulans* LW14 + protein fraction, *FJAPl* sterilised apple juice + *A. pullulans* LW14 + polysaccharide fraction, *FJAPP* sterilised apple juice + *A. pullulans* LW14 + protein fraction + polysaccharide fraction

Protein or polysaccharides from the biomass of *A. platensis* were not tested against fungi yet so they were chosen for the next research step. The growth of fungal biomass in apple juice inoculated with *A. pullulans* LW14 (FJA, Fig. 1a) continued for the whole period of cold storage (96 h). In all other cases, the quantity of fungal cells was significantly lower and did not exceed 10^5^ cfu/ml. In soft drinks with Spirulina (FJAS) or its protein fraction (FJAPt), the growth of fungal biomass stopped after 24 h of cold storage. Concentrations of fructose and glucose did not vary among tested variants at all time intervals (Fig. 1b, c). In the case of sucrose, its level was significantly lower in variants containing polysaccharides (FJAPl) and both isolated biomass fractions (FJAPP) while it was highest in the soft drink with added protein fraction (FJAPt) but it did not change in time in all experimental variants (Fig. 1d). *A. pullulans* prefers sucrose over other carbon sources for the production of pullulan [19]. Since exopolysaccharides are synthesised under stress conditions, i.e. limited oxygen source it is suspected that tested strain formed pullulan. Pullulan production was also observed when LW14 was cultivated on the rotary shaker prior to experiments. Soft drink with Spirulina (FJAS) demonstrated highest pH values and lowest total acidity (Fig. 1e, f). Highest total acidity was measured in FJAPt, while lowest pH was reported for the control sample—juice inoculated with fungal biomass (FJA). Therefore, it possible that Spirulina, particularly its protein fraction, inhibits fungal metabolism which translates to lower number of *A. platensis* LW14 cells.

### Antifungal Activity of Protein from Spirulina in Unpasteurised Apple Juice

Last research stage was carried out in unpasteurised apple juice. We added 0.72% of protein fraction which was previously demonstrated to hold mycostatic properties. It was noted that after 72 h, the number of LAB increased significantly and abruptly decreased after 96 h (Table 2) in unpasteurised juice (UJ) or juice inoculated with *A. pullulans* LW14. We did not detect any acetic acid bacteria, however, we could not exclude their presence. It would be necessary to pass samples through membrane filters which was not possible due to their cloudiness. In experimental variants containing Spirulina (UJAS) or its protein fraction (UJAP) the number of total fungi and total viable count were similar for the whole cold storage period (Table 2) which could confirm that antimicrobial properties of Spirulina are related to its protein fraction.

**Table 2:** The impact of Spirulina or its protein fraction on the native microflora of unpasteurised apple juice inoculated with *Aureobasidium pullulans* LW14

Experimental variant	Day	Total viable count [cfu/ml]	Total fungi [cfu/ml]	Lactic acid bacteria [cfu/ml]
UJ	0	5.20 ± 2.39 × 10^3b^	1.2 ± 0.34 × 10^3c^	0 ± 0^d^
	1	7.40 ± 2.70 × 10^3b^	4.12 ± 0.92 × 10^3c^	0 ± 0^d^
	2	7.40 ± 4.72 × 10^3b^	6.50 ± 1.61 × 10^3c^	1 ± 0^d^
	3	3.34 ± 1.11 × 10^4b^	4.34 ± 0.82 × 10^4c^	> 3 × 10^3a^
	4	5.40 ± 2.57 × 10^5b^	2.45 ± 0.29 × 10^5a^	2.36 ± 0.90 × 10^2b^
UJA	0	1.80 ± 0.84 × 10^5b^	1.56 ± 0.36 × 10^5c^	0 ± 0^d^
	1	3.80 ± 1.92 × 10^5b^	1.60 ± 0.89 × 10^5c^	0 ± 0^d^
	2	3.40 ± 2.51 × 10^5b^	1.80 ± 1.87 × 10^5c^	2 ± 1^d^
	3	3.20 ± 3.27 × 10^6b^	3.04 ± 1.08 × 10^6a^	> 3 × 10^3a^
	4	1.20 ± 0.22 × 10^7a^	3.70 ± 1.41 × 10^6a^	1.15 ± 1.00 × 10^2c^
UJAS	0	2.40 ± 1.14 × 10^5b^	1.56 ± 0.34 × 10^5c^	0 ± 0^d^
	1	5.40 ± 1.67 × 10^5b^	2.00 ± 1.00 × 10^5c^	0 ± 0^d^
	2	3.00 ± 2.35 × 10^5b^	1.60 ± 0.55 × 10^5b^	3 ± 4^d^
	3	1.40 ± 0.55 × 10^6b^	3.80 ± 1.92 × 10^5bc^	2 ± 1^d^
	4	3.00 ± 1.87 × 10^5b^	1.60 ± 1.34 × 10^5c^	3 ± 2^d^
UJAP	0	2.20 ± 0.84 × 10^5b^	1.54 ± 0.38 × 10^5c^	0 ± 0^d^
	1	2.00 ± 1.00 × 10^5b^	3.20 ± 0.84 × 10^5bc^	0 ± 0^d^
	2	2.40 ± 1.34 × 10^5b^	3.24 ± 1.52 × 10^5c^	3 ± 2^d^
	3	2.80 ± 1.92 × 10^6b^	2.20 ± 1.01 × 10^6ab^	2 ± 1^d^
	4	6.80 ± 4.65 × 10^5b^	9.00 ± 3.08 × 10^5bc^	1 ± 1^d^

*UJ* Unpasteurised apple juice, *UJA* unpasteurised apple juice + *A. pullulans* LW14, *UJAS* unpasteurised apple juice + *A. pullulans* LW14 + Spirulina, *UJAP* unpasteurised apple juice + *A. pullulans* LW14 + protein fraction

The same letters next to arithmetic means within columns indicate the lack of statistically significant differences at p < 0.0001, n = 5

**Fig. 2:**
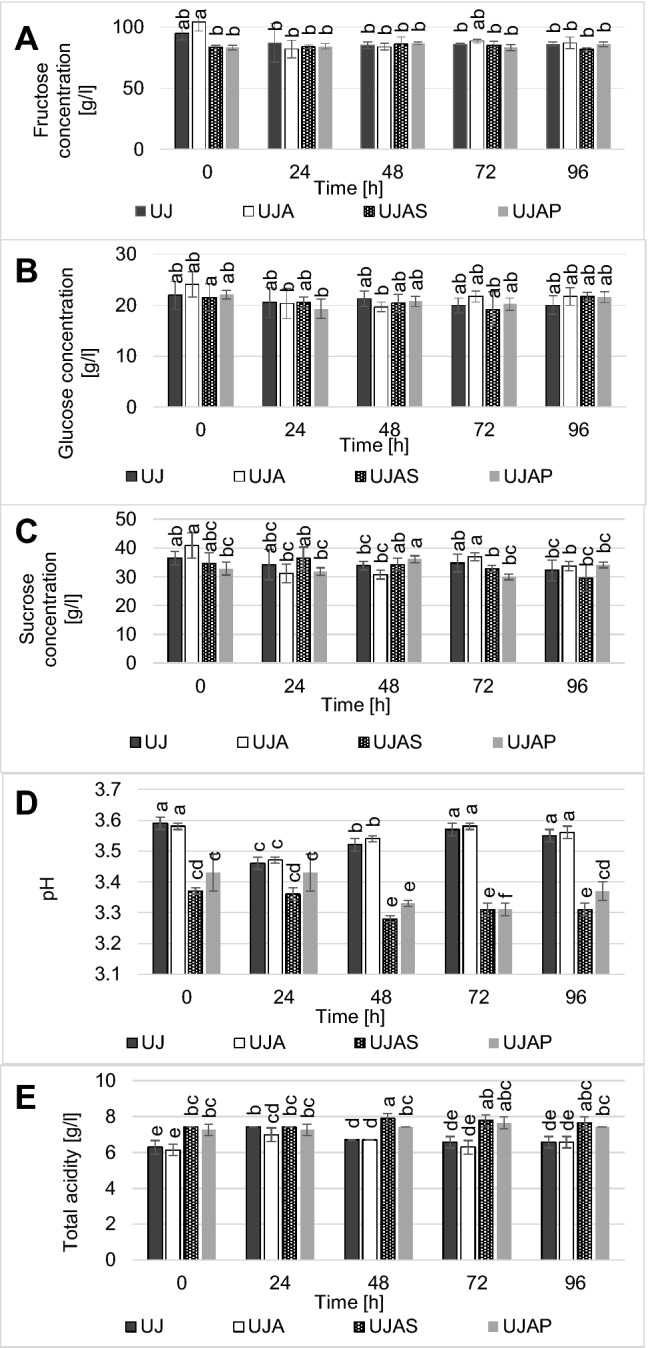
Microbiological, chemical and physical changes in unpasteurised apple juice fortified with protein Spirulina fraction: **a** Fructose concentration; **b** glucose concentration; **c** sucrose concentration; **d** pH; **e** total acidity expressed as malic acid. The same letters above columns indicate the lack of statistically significant differences at: p < 0.001 (2b), p < 0.0001 (2a; 2c–2e), n = 5. *UJ* unpasteurised apple juice, *UJA* unpasteurised apple juice + *A. pullulans* LW14, *UJAS* unpasteurised apple juice + *A. pullulans* LW14 + Spirulina, *UJAP* unpasteurised apple juice + *A. pullulans* LW14 + protein fraction

Fructose content decreased only in the juice inoculated with *A. pullulans* LW14 (UJA) but it remained constant in other tested variants (UJAS, UJAP, Fig. 2a). Glucose concentration did not change significantly for the whole cold storage and it was approximately even among all tested samples (Fig. 2b), while in the case of sucrose it decreased significantly only in UJA (Fig. 2c). Higher total acidy and lower pH was noted in UJAS and UJAP but at the same time total acidity decreased during cold storage (Fig. 2d, e).

The results of experiments carried out in unpasteurised apple juice with Spirulina (UJAS) or its protein fraction (UJAP) proved almost complete inhibition of lactic acid bacteria (LAB) (Table 2). It has been previously demonstrated that the presence of *A. platensis* biomass in yogurt stimulates the growth of probiotic LAB or the synthesis of lactic acid [20, 21] but its impact on LAB causing food spoilage was not examined. Based on findings reported in our paper it might be concluded that *A. platensis* could effectively inhibit the growth of LAB that reside in apples and protein fraction obtained from that cyanobacterium is mostly responsible for that effect. Drastic decrease of LAB in unpasteurised juice or juice inoculated with *A. pullulans* LW14 could be related to the extensive growth of other groups of microorganisms or secretion of their metabolites or enzymes which could cause damage of LAB cells. It was shown that some protein (nisin) could effectively inhibit growth of *Leuconostoc oenos* [22] or *Lactobacillus sakei* [23].

It was also noted that the presence of Spirulina or its protein fraction enhances the occurrence of colonies characteristic for *Metschnikowia pulcherrima* (green round colonies producing red pigment diffusing to the growth medium) in UJAS and UJAP variants, respectively. This yeast can remove iron from the environment and produces lytic enzymes like chitinase [24]. Most of the research involving that fungi focuses on its antifungal properties but some studies indicate that it might be effective against Gram-positive bacteria like *Bacillus subtilis* while it seems ineffective against some Gram-negative bacteria, i.e. *Pseudomonas aeruginosa* and *Asaia bogorensis* [25]. Therefore *M. pulcherrima* might inhibit growth of LAB.

It might be also concluded that there are some substances present in the biomass of *A. platensis* which induce the synthesis of organic acids because pH significantly decreased during the cold storage in the variant comprising of unpasteurised apple juice and Spirulina (Fig. 2d). In UJAP significant drop of pH was noted within 72 h but it increased again after 96 h. Therefore, it might be concluded that there are some other substances, except for protein which induce acid production in microorganisms. Changes of pH and total acidity indicate that at each day of cold storage, microbial consortia change their composition but it requires further research.

Our findings provide new directions for research. It is necessary to evaluate exactly which substances cause the inhibition of *Aureobasidium pullulans* and LAB and how they change microbial consortia of unpasteurised soft drinks during the cold storage. Then it is possible to widespread the application of those substances to other groups of food products for their preservation.
